# Personality and situational predictors of insider threat: a vignette study

**DOI:** 10.1080/00049530.2026.2628384

**Published:** 2026-03-01

**Authors:** Loch Forsyth, Tayla Ewens, Jeromy Anglim

**Affiliations:** School of Psychology, Deakin University, Geelong, Australia

**Keywords:** Personality, Dark Tetrad, insider threat, big five personality

## Abstract

**Objective:**

Insider threats pose a significant security risk, yet personality traits are rarely incorporated into detection and prevention efforts. This study offers the first systematic examination of how Dark Tetrad (i.e. Machiavellianism, narcissism, psychopathy, and sadism) and Big Five personality traits, together with situational motivators, predict the propensity to engage in three types of insider attacks.

**Method:**

Participants (*N* = 470; 71% female; age *M* = 35.94, *SD* = 12.70) completed measures of the Dark Tetrad (SD3, SSIS) and Big Five domains and facets (BFI-2), then responded to three counterbalanced vignettes depicting National Security Espionage, Information Technology Sabotage, and Fraud. For each vignette, participants rated their likelihood of engaging in the behaviour under baseline conditions and when (a) aggrieved, (b) facing termination, or (c) able to secure a financial gain.

**Results:**

Participants showed greater willingness to engage in fraud and information technology sabotage if they felt aggrieved or anticipated termination. Machiavellianism (*r* = .49), psychopathy (*r* = .50), and sadism (*r* = .53) showed strong positive associations with insider-attack propensity. Agreeableness (*r* = -.50) and conscientiousness (*r* = -.37) were the strongest Big Five predictors.

**Discussion:**

The findings indicate that antagonistic and exploitative personality traits, combined with situational pressures, meaningfully shape intentions to engage in insider misconduct.

## KEY POINTS

**What is already known about this topic:**

1. Insider threats can cause significant financial, operational, and security harm, yet most detection and prevention efforts focus on technical or organizational controls rather than individual differences.
2. Personality traits, particularly low agreeableness and low conscientiousness, are known predictors of workplace deviance more broadly.
3. Situational pressures such as perceived injustice, termination, and opportunities for financial gain can increase the likelihood of misconduct.

**What this study adds:**

1. This study provides the first systematic assessment of how Big Five traits and Dark Tetrad traits jointly relate to insider-attack intentions across multiple attack types.
2. Antagonistic and exploitative traits (psychopathy, Machiavellianism, and sadism) were the strongest dispositional predictors, while low agreeableness and low conscientiousness also meaningfully contributed.
3. Situational motivators, particularly feeling aggrieved or facing termination, substantially increased willingness to engage in insider misconduct, highlighting the value of integrating trait and context information in insider-risk programs.

Trusted insiders have been committing attacks on organizations for centuries (Finlay, 2010). The potential for trusted insiders to carry out an attack remains a critical vulnerability for contemporary organizations (Maasberg et al., 2015; McCormac et al., 2012). Insiders have access to data, systems, and resources that can be misused for purposes such as national security espionage, Information Technology sabotage, fraud, and theft of intellectual property (Band et al., 2006; Cappelli et al., 2009; Gelles, 2016; McCormac et al., 2012; Pfleeger et al., 2009). Intelligence agencies have labelled insider attacks as pervasive (ASIO, 2021), with the potential to effect organizations across all sectors (Silowash et al., 2012). Harmful outcomes of insider attacks include financial loss, operational impacts, reputational damage, and compromised national security (Cappelli et al., 2009; Gelles, 2016; McCormac et al., 2012; Silowash et al., 2012). Both case studies of insider attacks (for example, see McCormac et al., 2012; Whitty, 2018) and theoretical perspectives such as the Capability – Motive – Opportunity Model (Maasberg et al., 2015) highlight the role of dispositional tendencies and contextual factors. Identifying the personal and situational antecedents of insider attacks is therefore important for informing preventative actions, including candidate screening, training, and insider threat programs (for a review see Dalal et al., 2022).

Despite this importance, several gaps remain. First, although literature on the personality correlates of deviant behaviour is broad (Lee et al., 2019; Pletzer et al., 2019, 2020), recent reviews note that empirical research has not systematically examined how personality traits relate to insider-attack intentions (Harms et al., 2022; Marbut & Harms, 2024; Ruohonen & Saddiqa, 2025). Indeed, after conducting a systematic review of the psychology of insider threat, Ruohonen and Saddiqa (2025) noted that they found “no relevant papers published in psychology journals” (p. 206). Second, there is limited evidence on how contextual factors influence insider-attack propensity and how these factors interact with personality traits (for a recent exception, see Idensohn et al., 2026). Third, while case analyses provide insights into individual incidents, they do not permit systematic examination of personality correlates across scenarios. Accordingly, the current study aimed to identify the personality and situational factors that heighten the likelihood of engaging in an insider attack.

## Personality

Personality traits represent stable individual differences in how people think, feel, and behave (Roberts & Yoon, 2022). Drawing on lexical and factor analytic traditions, the Big Five traits (i.e., neuroticism, extraversion, openness, agreeableness, and conscientiousness) have emerged as the dominant framework for describing broad dispositional tendencies (Condon, 2017; Costa & McCrae, 1995; John & Srivastava, 1999; Markon et al., 2005). However, some researchers have argued that the Big Five does not fully capture socially aversive or exploitative tendencies that are relevant to predicting deviant behaviour (e.g., Jonason & O’Connor, 2017; Lee et al., 2003, 2008). The Dark Tetrad – Machiavellianism, narcissism, psychopathy, and sadism – has emerged as a complementary set of traits capturing these tendencies (Blötner et al., 2022; Paulhus et al., 2021; Turi et al., 2022). From the perspective of the Big Five, these traits partly reflect low agreeableness and, to a lesser extent, low conscientiousness, but they also capture additional variance linked to manipulativeness, callousness, and enjoyment of others’ suffering (Costa & McCrae, 1995; Gaughan et al., 2012). Evidence suggests that the Dark Tetrad can sometimes outperform broad traits in predicting deviant and antisocial behaviour (Howard & Van Zandt, 2020).

Personality traits are typically conceptualized hierarchically, with broad domains each represented by narrower facets that offer greater specificity (Anglim & O’Connor, 2019; John & Srivastava, 1999). Major hierarchical models include the NEO PI-R (Costa & McCrae, 1992), the BFI-2 (Soto & John, 2017), and the Big Five Aspects Scale (DeYoung et al., 2007). Similarly, researchers have also proposed a broad “Dark Core” underlying the Dark Tetrad traits (Bader et al., 2021; Moshagen et al., 2020), while others emphasize examining the individual traits because they show distinct predictive profiles and nomological networks. Although there is no consensus about the optimal number of personality facets, facet-level assessment often improves prediction of criteria and provides better theoretical insights (Anglim et al., 2018, 2020; Pletzer et al., 2020).

## Personality and the motivators of insider attacks

While research has not yet comprehensively examined personality correlates of insider attacks, the broader literature on deviant behaviour in the workplace provides insights. Meta-analytic evidence shows that lower agreeableness and lower conscientiousness are the strongest Big Five correlates of counterproductive work behaviour, with neuroticism showing a modest positive association (Pletzer et al., 2019). Traits related to self-promotion, social aggressiveness, and emotional coldness also predict deviant behaviour (Ellen et al., 2021; Jones & Kavanagh, 1996; Maasberg et al., 2015; Paulhus & Williams, 2002; Stoica, 2021). Research further highlights the importance of facet-level analysis, which often yield more precise predictions (Anglim et al., 2018; Pletzer et al., 2019, 2020). Finally, meta-analyses and reviews show that personality correlates differ across types of deviance: interpersonal deviance is more strongly associated with low agreeableness and organizational deviance is more strongly associated with low conscientiousness (Pletzer et al., 2019; Wiernik & Ones, 2018).

Theoretical perspectives help clarify why dark traits may predispose individuals to insider attacks. Both moral disengagement and evolutionary accounts suggest that tendencies towards personal gain, exploitation, and diminished social responsibility increase the likelihood of aggression and norm violation (Kapoor et al., 2021; Roeser et al., 2016). These dispositions map closely onto the Dark Tetrad traits and may manifest in the workplace as willingness to instigate or carry out an insider attack.

Although research on trait–situation interactions within insider-threat contexts is relatively limited (Wille et al., 2023), some initial studies address this gap. Notably, Idensohn et al. (2026) examined the role of Machiavellianism in conjunction with situational factors drawn from the Fraud Triangle, namely pressure, opportunity and rationalization, in predicting intentions to commit malicious insider behaviour. They found that Machiavellianism predicted all three situational perceptions but was most strongly related to rationalization. Complementing this work, case studies highlight a range of situational factors that can heighten attack motivation, including perceived injustice, organizational mistreatment, and opportunities for financial gain (Gelles, 2016; Maasberg et al., 2015; Moore et al., 2008; Silowash et al., 2012). This aligns with Trait Activation Theory, which holds that dispositional tendencies are more likely to emerge when situational cues signal that certain behaviours are possible or advantageous (Tett & Burnett, 2003). These effects can vary across narrower facets of a trait, underscoring the value of examining both broad and specific personality dimensions (Wille et al., 2023). Experimental work also suggests that Machiavellianism may be particularly sensitive to situations that allow exploitation (Bereczkei & Czibor, 2014). Collectively, the integration of trait activation, evolutionary perspectives, and moral disengagement theories provides a framework for understanding how personality traits and situational motivators jointly influence insider-attack intentions.

## The current study

The current study examined how personality traits and situational motivators predict the propensity to engage in insider attacks. We assessed Big Five domains and facets, as well as Dark Tetrad traits, and evaluated their ability to predict attack intentions across a series of vignettes. Each vignette depicted one of three insider-attack contexts: national security espionage, fraud, or information technology sabotage. For each scenario, participants rated their likelihood of engaging in the behaviour under baseline conditions and when facing three motivators: imminent termination, feeling aggrieved, and the opportunity for financial gain. This design allowed us to evaluate both the shared and unique contributions of Big Five traits and Dark Tetrad traits, and to compare prediction across broad and facet-level representations of personality.

## Method

Data, analysis scripts, and study materials are available on the OSF (Forsyth et al., 2026) at https://osf.io/x5gpb/?view_only=cee39369594e4d8691aa1dd345d4122e

### Participants and procedure

Participants were recruited via email invitations and online social media posts. Participation was voluntary and no incentives were offered. The study received ethics approval from the first author’s human research ethics committee (HEAG-H 214–2020). Participants first completed the personality measures, followed by the three vignettes presented in random order.

The final sample consisted of 470 participants (71.3% Female, 27.4% Male, 1.3% other) with a mean age of 35.94 years (*SD* = 12.70, range: 18 to 80). Most participants lived in Australia (67%) and were in paid employment (77%). The final cleaned sample was obtained after deleting five participants because they (a) completed the survey so quickly that the participant would not have sufficient time to read and comprehend the items (i.e., less than 6 minutes), or (b) showed evidence of non-conscientious responding on the personality items (i.e., within-person standard deviation in item responses less than 0.3 on the BFI-2) (for discussion, see Curran, 2016).

### Materials

#### Personality

Of the Dark Tetrad, Machiavellianism, psychopathy, and narcissism were measured using the 27-item Short Dark Triad (SD3; Jones & Paulhus, 2014) and sadism was measured using the 10-item Short Sadistic Impulse Scale (SSIS; O’Meara et al., 2011). The Big Five domains, each with three nested facets, were measured using the 60-item Big-Five-Inventory-2 (BFI-2; Soto & John, 2017). All items were rated on a scale from *1 = Disagree Strongly* to *5 = Agree Strongly*. Relevant items were reversed, and total mean scores calculated for each trait. We also computed an overall Dark Tetrad composite as the mean of the four Dark Tetrad scale scores. This composite was used for pragmatic, comparative purposes to evaluate the predictive utility of an aggregate dark-trait index relative to its component traits, rather than to imply that the Dark Tetrad reflects a unidimensional construct.

#### Insider threat vignettes

Three vignettes were developed to represent common insider attack types: (1) national security espionage, (2) fraud, and (3) information technology sabotage. These attack types were selected based on reviews of publicly available case studies and prior research. The vignettes were piloted with industry threat experts to ensure that each scenario reflected contemporary insider-risk contexts. Their length and structure were designed to align with established vignette-based methodologies (Baughman et al., 2014; Forsyth et al., 2021; Telwatte et al., 2017). Each vignette described a workplace scenario in which participants were asked to indicate whether they would engage in the specified insider behaviour (see OSF Supplement). The vignettes were created specifically for this study, as no existing materials covered multiple insider-attack types.

After reading each vignette, participants responded to four items assessing their willingness to engage in the behaviour: (a) their baseline likelihood, and their likelihood if (b) facing imminent termination, (c) able to secure a large financial gain, or (d) feeling angry and betrayed by the organization (aggrieved). Responses were provided on a scale from 1 (Definitely Not) to 5 (Definitely Would). Participants therefore provided 12 ratings in total (three vignettes by four conditions). An overall propensity score was computed by averaging the 12 items, and additional composite scores were created for each individual attack type.

### Data analytic approach

All data analyses were conducted using R 4.5 (R Core Team, 2021). Personality correlates of intentions to engage in insider attacks were examined using Pearson correlations, with emphasis placed on the overall pattern of associations. To assess the relative contribution of different personality domains, a series of regression models with varying predictor sets were estimated and compared using adjusted *R*^2^.

## Results

### Propensity across scenarios and motivational conditions

Descriptive statistics for insider-attack propensity are presented in Table 1. Participants showed the highest willingness to engage in fraud, followed by national security espionage and information technology sabotage. Consistent with insider attacks being highly deviant behaviour, means levels were low overall (1.7 on a 1 to 5 scale). Termination and feeling aggrieved increased the propensity to engage in fraud and information technology sabotage but did not meaningfully affect intentions to commit national security espionage.

**Table 1.** Descriptive statistics for insider threat propensity items across attack types for baseline propensity and propensity with motivating factors.

	Scenarios			
	NSE	Fraud	IT Sabotage	Overall
	*M* (*SD*)	*M* (*SD*)	*M* (*SD*)	*M* (*SD*)
Motivator				
Baseline	1.89 (1.07)	1.70 (1.12)	1.26 (0.70)	1.62 (0.71)
Financial	1.78 (1.09)	1.78 (1.18)	1.40 (0.89)	1.65 (0.87)
Termination	1.64 (0.99)	2.13 (1.40)	1.61 (1.08)	1.79 (0.90)
Aggrieved	1.70 (1.02)	2.00 (1.25)	1.54 (1.02)	1.74 (0.87)
Overall	1.75 (0.88)	1.90 (1.16)	1.45 (0.83)	1.70 (0.78)

*Note*. NSE = national security espionage; IT = information technology.

### Correlations

Correlations between demographics, broad personality traits, and insider-attack propensity are shown in Table 2. The strongest personality correlate of insider-attack propensity was the overall Dark Tetrad composite (*r* = .57). Among the Big Five, agreeableness (*r =* −.50) and conscientiousness (*r* = −.37) showed the largest associations with lower attack propensity. As expected, the Dark Tetrad composite correlated strongly and negatively with agreeableness (*r =* −.68). Males and younger adults tended to score lower on agreeableness, higher on overall Dark Tetrad, and reported a greater willingness to engage in an insider attack. With a few exceptions, correlations were broadly similar across scenarios and motivators, although personality associations were generally weaker for national security espionage.

**Table 2.** Descriptive statistics and correlations.

Variable	⍺	*M*	*SD*	1	2	3	4	5	6	7	8	9	10	11	12	13	14	15
Insider Threat																		
1. Overall	.92	1.70	0.78															
2. NSE	.86	1.75	0.88	.73														
3. Fraud	.95	1.90	1.16	.87	.41													
4. IT Sabotage	.92	1.45	0.83	.83	.42	.63												
5. Baseline	.55	1.62	0.71	.90	.71	.76	.71											
6. Financial	.77	1.65	0.87	.95	.68	.84	.78	.87										
7. Termination	.66	1.79	0.90	.94	.66	.84	.78	.76	.83									
8. Aggrieved	.69	1.74	0.87	.93	.67	.80	.80	.74	.83	.87								
Predictors																		
9. Age		35.22	12.70	−.30	−.16	−.30	−.25	−.24	−.31	−.29	−.27							
10. Male		0.27	0.45	.14	.02	.15	.16	.10	.15	.12	.13	.04						
11. Neuroticism	.90	2.99	0.85	.12	.11	.10	.08	.07	.08	.14	.15	−.21	−.22					
12. Extraversion	.83	3.26	0.68	−.07	−.07	−.06	−.03	−.05	−.07	−.07	−.06	.15	.06	−.35				
13. Openness	.79	3.85	0.59	−.02	−.08	.03	−.02	−.05	−.02	−.02	−.01	.03	.06	−.03	.21			
14. Agreeableness	.84	3.74	0.68	−.50	−.29	−.45	−.47	−.44	−.49	−.46	−.47	.32	−.20	−.27	.09	.02		
15. Conscientiousness	.88	3.63	0.75	−.37	−.25	−.32	−.32	−.32	−.37	−.32	−.34	.27	−.19	−.30	.27	−.03	.47	
16. Dark Tetrad overall	.81	2.33	0.52	.57	.31	.53	.55	.52	.57	.51	.53	−.25	.27	.03	.17	.08	−.68	−.36

*Note. N = 470*. NSE = national security espionage; IT = information technology. Male was coded 0 = Female/Other, 1 = Male. Absolute correlations greater than or equal to .10, .12, and .16 are significant at .05, .01, and .001 (two-tailed) respectively.

To further refine these patterns, correlations between personality facets, individual Dark Tetrad scales, and attack propensity were examined (see Table 3; a complete correlation matrix of 35 variables is provided in the online supplement). Facets of Big Five agreeableness showed comparable correlations with attack propensity. In contrast, conscientiousness facets varied substantially, with responsibility showing the strongest association, followed by productiveness and order. Among the Dark Tetrad traits, Machiavellianism, psychopathy, and sadism were all strongly and positively correlated with insider-attack propensity, whereas narcissism showed a much weaker relationship.

**Table 3.** Correlations between personality facets, dark tetrad scales and insider threat scales.

			Scenarios			Motivators			
	⍺	Overall	NSE	Fraud	ITS	Baseline	Financial	Termination	Aggrieved
**Big Five**									
N1. Anxiety	.78	.01	.04	.01	−.02	−.02	−.03	.04	.05
N2. Depression	.79	.16	.13	.13	.12	.10	.12	.17	.17
N3. Emotional Volatility	.84	.14	.11	.13	.10	.09	.11	.15	.17
E1. Sociability	.82	−.06	−.05	−.06	−.04	−.05	−.08	−.06	−.04
E2. Assertiveness	.76	.08	−.02	.10	.09	.08	.07	.06	.07
E3. Energy Level	.62	−.19	−.12	−.19	−.15	−.17	−.17	−.19	−.18
O1. Intellectual Curiosity	.66	.05	−.05	.10	.05	.04	.06	.03	.05
O2. Aesthetic Sensitivity	.67	−.03	−.05	.00	−.04	−.08	−.05	.01	−.01
O3. Creative Imagination	.73	−.06	−.09	−.03	−.04	−.05	−.04	−.07	−.06
A1. Compassion	.65	−.42	−.25	−.37	−.40	−.40	−.42	−.36	−.37
A2. Respectfulness	.68	−.41	−.25	−.35	−.39	−.33	−.38	−.39	−.40
A3. Trust	.73	−.44	−.25	−.41	−.39	−.36	−.42	−.41	−.42
C1. Organization	.81	−.21	−.15	−.17	−.19	−.19	−.21	−.18	−.20
C2. Productiveness	.76	−.32	−.22	−.29	−.27	−.28	−.32	−.29	−.30
C3. Responsibility	.73	−.43	−.29	−.38	−.37	−.38	−.43	−.38	−.39
**Dark Tetrad**									
Machiavellianism	.81	.49	.27	.48	.43	.42	.49	.46	.45
Narcissism	.73	.29	.14	.27	.28	.27	.29	.24	.26
Psychopathy	.75	.50	.30	.45	.48	.46	.51	.45	.47
Sadism	.90	.53	.28	.47	.54	.48	.53	.47	.49

*Note. N =* 470. NSE = national security espionage; ITS = information technology sabotage. Absolute correlations greater than or equal to .10, .12, and .16 are significant at .05, .01, and .001 (two-tailed) respectively.

### Regression models

To evaluate the predictive contribution of different personality frameworks, we estimated a series of regression models predicting overall insider-attack propensity (see Table 4). The models assessed the following predictor sets: (1) Big Five domains, (2) 15 Big Five facets, (3) an overall Dark Tetrad composite, (4) four Dark Tetrad scales, and (5) a combined model including Big Five domains and the four Dark Tetrad scales.

**Table 4.** Regression models predicting overall insider threat from different sets of personality predictors.

	M1 β (SE)	M2 β (SE)	M3 β (SE)	M4 β (SE)	M5 β (SE)
Neuroticism	−0.04 (0.04)				0.01 (0.04)
Extraversion	0.01 (0.04)				−0.07 (0.05)
Openness	−0.03 (0.04)				−0.04 (0.04)
Agreeableness	−0.43* (0.05)				−0.11* (0.06)
Conscientiousness	−0.18* (0.05)				−0.12* (0.05)
N1. Anxiety		−0.03 (0.06)			
N2. Depression		0.03 (0.06)			
N3. Emotional Volatility		−0.05 (0.06)			
E1. Sociability		−0.02 (0.05)			
E2. Assertiveness		0.09 (0.05)			
E3. Energy Level		−0.01 (0.05)			
O1. Intellectual Curiosity		−0.01 (0.05)			
O2. Aesthetic Sensitivity		0.01 (0.04)			
O3. Creative Imagination		−0.04 (0.05)			
A1. Compassion		−0.11 (0.05)			
A2. Respectfulness		−0.05 (0.06)			
A3. Trust		−0.24* (0.06)			
C1. Organization		0.07 (0.05)			
C2. Productiveness		−0.08 (0.06)			
C3. Responsibility		−0.27* (0.06)			
Tetrad Overall			0.57* (0.04)		
Machiavellianism				0.24* (0.05)	0.20* (0.05)
Narcissism				−0.01 (0.04)	0.08 (0.05)
Psychopathy				0.17* (0.06)	0.07 (0.06)
Sadism				0.28* (0.05)	0.22* (0.06)
Adjusted *R*^2^	0.27	0.28	0.33	0.34	0.37
Adjusted *R*	0.52	0.53	0.57	0.59	0.61

*Note*. Coefficients represent standardized coefficients with standard error in parentheses. Adjusted r-squared allows for comparison of variance explained across models. Adjusted multiple *R* permits more direct comparison with bivariate correlations.

*p < .05.

Several key findings emerged. First, using 15 Big Five facets (adjusted *R* = .53) did not meaningfully improve prediction beyond the Big Five domains (adjusted *R* = .52). In the domain-level model, the strongest predictors were low agreeableness followed by low conscientiousness. In the facet-level model, low trust and low responsibility were the only significant predictors.

Second, the four Dark Tetrad scales (adjusted *R* = .59) provided only a modest improvement in prediction over the overall Dark Tetrad composite (adjusted *R* = .57). This reflected the fact that three of the four Dark Tetrad traits, psychopathy, sadism, and Machiavellianism, showed similar bivariate correlations with insider-attack propensity and comparable regression coefficients.

Third, combining Big Five domains with the Dark Tetrad scales (adjusted *R* = .61) improved prediction relative to the Big Five alone, although the incremental gain over the Dark Tetrad scales alone was small. Despite overlap between the Big Five and Dark Tetrad frameworks, the combined model highlighted Machiavellianism, sadism, low agreeableness, and low conscientiousness as the significant predictors.

## Discussion

The current study provides a comprehensive examination of how personality traits and situational motivators predict the propensity to engage in insider attacks. Several key findings emerged. First, among the Big Five traits, low agreeableness and low conscientiousness were the strongest predictors of attack propensity. Second, within the Dark Tetrad traits, Machiavellianism, psychopathy, and sadism all emerged as strong predictors of intention to engage in an insider attack. Third, participants indicated greater willingness to carry out an insider attack when they felt aggrieved or anticipated termination. Together, these findings highlight the joint influence of dispositional tendencies and situational pressures on insider-attack intentions and carry theoretical and practical implications.

### Personality and insider attack

The pattern of personality correlates was consistent with assumptions derived from moral disengagement and evolutionary perspectives. A cluster of inter-related traits reflecting antagonism, dishonesty, rule violation, emerged as the strongest predictors of insider-attack propensity. Among the Dark Tetrad traits, psychopathy and sadism, which were strongly correlated with each other (*r* = .71), showed the largest associations across scenarios. Sadism was most strongly related to information technology sabotage, which may reflect the appeal of inflicting harm and observing its consequences. Machiavellianism also showed consistent associations across attack types, consistent with the results of Idensohn et al. (2026). Prior work suggests that individuals high in psychopathy and Machiavellianism are motivated more by instrumental gain than by ego-reinforcement (Jones & Paulhus, 2014), which aligns with the present findings. Future work could further refine these associations using multidimensional models of Machiavellianism, such as Monaghan et al’.s (2020) distinction between cynical worldviews and tactical manipulation.

The strongest dispositional predictors of insider-attack intentions therefore reflected tendencies towards antagonism and opportunistic exploitation. This aligns with broader evidence that individuals high in callous–manipulative traits are more willing to violate norms for personal advantage (Baughman et al., 2014), including engaging in workplace deviance (Anglim et al., 2018; Jonason & O’Connor, 2017; Pletzer et al., 2019) and unethical behaviour (Lee et al., 2008). These effects were most pronounced in scenarios involving potential financial gain, consistent with trait activation accounts in which dispositional tendencies towards exploitation are more likely to manifest when situational cues make such behaviour rewarding or advantageous (Idensohn et al., 2026; Jonason & O’Connor, 2017). More generally, the findings highlight the need to consider how antagonistic traits interact with contextual triggers to shape misconduct.

Conscientiousness, particularly its responsibility facet, also emerged as a notable predictor. Whereas low agreeableness and Dark Tetrad traits capture interpersonal antagonism, conscientiousness reflects a distinct dimension associated with rule-following and dependability. Its importance aligns with evidence that low agreeableness is more predictive of interpersonal deviance, whereas low conscientiousness more strongly predicts organizational deviance (Pletzer et al., 2019). In the context of insider attacks, this distinction is meaningful, as many attack types directly involve neglecting duties, breaching procedures, or deliberately undermining organizational functioning.

Overall, these findings contribute to an understanding of how dispositional and situational factors jointly shape insider threat intentions. Personality correlates were broadly similar across motivational contexts, suggesting that the situational triggers examined, such as grievance or financial pressure, may amplify underlying dispositional tendencies rather than fundamentally change their direction. By contrast, national security–motivated scenarios showed weaker personality associations, consistent with the idea that ideologically framed threats may rely more on contextual, social, or institutional influences than on individual differences alone. This pattern aligns with evidence from the counterproductive work behaviour literature, which shows that while the broad pattern of personality–behaviour associations is fairly robust, the relative importance of specific traits depends on the nature of the behaviour. Notably, agreeableness-related traits tend to be more strongly related to interpersonal deviance and conscientiousness-related traits more strongly linked to organizational deviance (Berry et al., 2007). Many classic malicious insider threat scenarios involve organizationally directed harm, for which low conscientiousness and antagonistic, self-interested traits may be especially relevant. From a theoretical perspective, these findings are broadly consistent with integrative accounts of insider risk, such as those drawing on the fraud triangle (e.g., Idensohn et al., 2026), insofar as rationalization overlaps conceptually with dispositional tendencies that normalize manipulation, rule-bending, and self-interest. At the same time, pressure and opportunity, often treated as situational features, are subjectively experienced and likely shaped in part by personality. Future research would benefit from integrative models that more explicitly examine how personality influences the perception, interpretation, and impact of situational pressures and opportunities over time.

### Practical implications

While acknowledging that the data is based on self-reported intentions to engage in insider attacks and not actual behaviour, the findings nevertheless have several potential practical implications. Well-validated personality measures may assist in identifying applicants who have a greater propensity to engage in insider misconduct, particularly when combining hierarchical measures of the Big Five with measures of dark traits. In high-stakes settings, procedures to reduce faking should be incorporated into assessment procedures (Dunlop et al., 2025; Robie et al., 2021). These may include warning applicants not to fake (Mcfarland, 2003), use of measures with subtle or less evaluative items (Wood et al., 2022), and combining ipsative and normative response formats (Bowen et al., 2002; Salgado et al., 2015). Organizations may also benefit from supplementing non-self-report inventories with alternative strategies such as structured psychological interviews, reference checks, and check on past behaviour (e.g., criminal records and employee history). There is also the potential to combine recent developments in artificial intelligence and the popularity of automated video interviews to improve such assessments at scale (Hickman et al., 2022, 2024). When embedded within a broader, organization-specific insider-threat program, such practices may strengthen overall security culture. Although these approaches are relevant across sectors, they are likely to be especially useful for organizations that represent high-value targets because of their intellectual property, financial assets, or national-security relevance. Finally, the results reinforce the ongoing importance of monitoring situational triggers. Employees who feel aggrieved or anticipate termination may be at elevated risk of engaging in harmful behaviour, underscoring the value of ongoing organizational awareness and early intervention.

### Limitations and future research

Several limitations should be noted. First, the study examined insider-attack propensity using vignettes rather than observing actual behaviour, and this limits direct generalizability to real-world events. That said, objective or observer-reported indicators of insider attacks are difficult to obtain, often inaccurate, and may fail to capture covert or infrequent behaviours. Vignettes offer a practical alternative because they allow controlled manipulation of contextual motivators, align with calls for research examining trait–situation interactions, and make it feasible to collect the large samples needed to detect differential personality correlates. Future research could extend this work by linking personality data with real organizational outcomes where possible, by examining a broader range of contextual triggers and attack types, and by further refining vignette approaches to capture more diverse insider-threat scenarios.

Second, endorsement levels for intentions to engage in insider threats were low overall (*M* = 1.70 on a 1–5 scale), as expected given the highly malicious nature of the behaviours assessed. This reflects the low base-rate nature of the phenomenon rather than a measurement flaw, though it may attenuate prediction relative to more common forms of deviance. Despite this constraint, a theoretically coherent and relatively strong pattern of associations emerged in predicting insider-threat intentions.

## Conclusion

The current study offers a comprehensive examination of how personality traits relate to insider-attack propensity. The findings highlight the value of considering both dispositional tendencies and contextual motivators when assessing insider risk, and they clarify how traits from two major frameworks, the Big Five and the Dark Tetrad, may contribute to attack intentions. More broadly, the results highlight the potential role of personality assessment in employee selection and ongoing monitoring as part of organizational efforts to reduce insider-related harm and strengthen security practices.

## Supplementary Material

Complete correlation matrix

Insider Threat Vignettes

## Data sharing statement

Data, analysis scripts, and study materials are available on the OSF at https://osf.io/x5gpb/?view_only=cee39369594e4d8691aa1dd345d4122e.

## Open scholarship

This article has earned the Center for Open Science badges for Open Data and Open Materials through Open Practices Disclosure. The data and materials are openly accessible at https://osf.io/x5gpb/?view_only=cee39369594e4d8691aa1dd345d4122e.
