# Supplementary material for: Personality and situational predictors of insider threat: a vignette study
Source: Aust J Psychol. 2026 Mar 1;78(1):2628384. doi: 10.1080/00049530.2026.2628384 (PMC12951658; doi:10.1080/00049530.2026.2628384)
Supplement: Insider Threat Vignettes [file RAUP_A_2628384_SM5583.docx]

# Online Supplement

# Insider Threat Vignettes

Each of the three insider threat vignettes developed for the present study represented an insider attack type, selected after being identified as three of the most widespread and pervasive insider attacks through a review of the research and publicly available insider attacks (see Ewens, 2021; Gelles, 2016). Namely, these attack types pertain to 1: *National Security Espionage,* 2: *Fraud,* and 3: *Information Technology Sabotage*. Vignette length and arrangement was developed to be consistent with similar vignette-based methodologies from previous peer reviewed research (Baughman et al., 2014; Forsyth et al., 2021). To ensure the vignettes were representative of modern day insider attacks occurring across industries and sectors, vignette’s underwent piloting with a range of industry threat experts and inter-rater reliability for each achieved.

As noted in the methods section, after reading each vignette participants indicated their propensity to engage in the behaviour just described (“*Please indicate the likelihood that you would carry out the above behaviour”*). Following, the same question was asked with the inclusion of the motivational factors of *termination, financial gain,* and/or *aggrievement* towards an organisation (e.g., *“Please indicate the likelihood that you would carry out the above behaviour if you could make a large financial gain*”) to determine if these motivational elements influenced propensity to engage in the described behaviours. The motivational factors pertained to the most salient motivating factors as documented within the existing insider threat literature outlined in the introduction which include three distinct categories related to *organisational (termination)*, *individual (aggrievement),* and *financial (financial gain)* motivations. Each insider threat vignette and the corresponding items are presented below.

## National Security Espionage

As part of your job your employer is paying for you to undergo specialised study at a university located overseas. This opportunity allows you to study with a number of well-known and internationally regarded experts on a cutting edge topic related to your work. You decide to accept the opportunity, and once study commences you find yourself working on a project that involves classified information. You are asked to keep this information confidential. You agree and as requested, sign an official contract to document your agreement. On completion of the study and on return to your own country, your workplace asks you to share this classified information with them, assuring you that doing so is vital to ensuring National Security.

## Financial Gain

You have been working at a new organisation for several months. Your six-month performance review is approaching, and you are aware that you will not meet several of your performance targets. As an incentive, employees receive a 20% pay increase for exceeding their performance targets. Having not met your performance targets, you will not be eligible for a pay increase. Prior to your performance review, you stumble across an electronic file available on the company’s network called ‘Performance Feedback’ containing all employee performance information. You realise that the file can be edited, and you can anonymously modify your own feedback rating to improve your score – ensuring a 20% pay increase. On doing this, the pay increase will automatically be approved and then paid to you.

## Information Technology Sabotage

As part of your role, you are allowed to create, update, and delete content on the company website. Over the last month, you along with your team have been asked by your employer to make a major update to their website. Due to your team being absent, you were required to complete the majority of the work. Your company was very happy with the improvements to the website. On completion you received zero acknowledgement or reward from your employer for your significant individual efforts in your teams absence. You become aware of a location on the dark web where you could anonymously post the access codes to your company’s internal network. Doing so would allow strangers access to the company’s confidential client information and the company website where they could freely delete information or add harmful content, effectively sabotaging the organisation.

## Vignette Questions

Each vignette was followed by the following four questions:

**Q1.** Please indicate the likelihood that you would carry out the above behaviour.

**Q2.** Please indicate the likelihood that you would carry out the above behaviour if you could make a large financial gain.

**Q3.** Please indicate the likelihood that you would carry out the above behaviour if you were aware you were to be unfairly terminated in the next six months.

**Q4.** Please indicate the likelihood that you would carry out the above behaviour if you felt angry and betrayed by something you held the organisation responsible for.
